# KMT2A promotes melanoma cell growth by targeting hTERT signaling pathway

**DOI:** 10.1038/cddis.2017.285

**Published:** 2017-07-20

**Authors:** Changlin Zhang, Chen Song, Tianze Liu, Ranran Tang, Miao Chen, Fan Gao, Binyi Xiao, Ge Qin, Fen Shi, Wenbin Li, Yixin Li, Xiaoyan Fu, Dingbo Shi, Xiangsheng Xiao, Lan Kang, Wenlin Huang, Xiaojun Wu, Bing Tang, Wuguo Deng

**Affiliations:** 1 Sun Yat-sen University Cancer Center; State Key Laboratory of Oncology in South China; Collaborative Innovation Center of Cancer Medicine, Guangzhou, China; 2 Department of Oncology, The Second Affiliated Hospital of Dalian Medical University; Institute of Cancer Stem Cell, Dalian Medical University, Dalian, China; 3 Department of Burn and Plastic Surgery, The First Affiliated Hospital, Sun Yat-sen University, Guangzhou, China; 4 State Key Laboratory of Targeted Drug for Tumors of Guangdong Province, Guangzhou Double Bioproduct Inc., Guangzhou, China

## Abstract

Melanoma is an aggressive cutaneous malignancy, illuminating the exact mechanisms and finding novel therapeutic targets are urgently needed. In this study, we identified KMT2A as a potential target, which promoted the growth of human melanoma cells. KMT2A knockdown significantly inhibited cell viability and cell migration and induced apoptosis, whereas KMT2A overexpression effectively promoted cell proliferation in various melanoma cell lines. Further study showed that KMT2A regulated melanoma cell growth by targeting the hTERT-dependent signal pathway. Knockdown of KMT2A markedly inhibited the promoter activity and expression of hTERT, and hTERT overexpression rescued the viability inhibition caused by KMT2A knockdown. Moreover, KMT2A knockdown suppressed tumorsphere formation and the expression of cancer stem cell markers, which was also reversed by hTERT overexpression. In addition, the results from a xenograft mouse model confirmed that KMT2A promoted melanoma growth via hTERT signaling. Finally, analyses of clinical samples demonstrated that the expression of KMT2A and hTERT were positively correlated in melanoma tumor tissues, and KMT2A high expression predicted poor prognosis in melanoma patients. Collectively, our results indicate that KMT2A promotes melanoma growth by activating the hTERT signaling, suggesting that the KMT2A/hTERT signaling pathway may be a potential therapeutic target for melanoma.

Melanoma is one of the most deadly cutaneous malignancies and increases in occurrence in the past several decades.^1, 2, 3, 4^ Currently, there may be one million melanoma patients in the United States. Up to 20% of the patients will develop metastatic tumors eventually, and the 5-year survival rate of them is <5% after the occurrence of metastasis.^5^ In recent years, improved knowledge of the pathophysiology of melanoma and a better understanding of the role of the immune system in tumor control have led to the development and application of several immunotherapies.^6^ Monoclonal antibodies against different immune checkpoints have revolutionized the treatment of metastatic and unrespectable melanoma. Ipilimumab and pembrolizumab have been shown to target cytotoxic T-lymphocyte antigen 4^7^ and programmed cell death protein 1,^8^ respectively, whereas vemurafenib targets BRAF signaling pathway.^9^ These therapies have prolonged the overall survival (OS) in patients with advanced melanoma. However, fair proportions of melanomas are BRAF wild type, NRAS-mutant or TERT-mutant, and hence are insensitive to these vemurafenib.^10, 11^ Also, metastatic melanomas still need good treatment options, as the underlying mechanisms of melanoma progression and metastasis are not well acknowledged.^12^ Therefore, it is crucial to discover and identify potential key players in melanoma tumorigenesis for the development of novel cancer therapeutics.

Lysine methyltransferase 2A (KMT2A), also known as mixed-lineage leukemia (MLL) or acute lymphoblastic leukemia 1 (ALL-1), is a transcriptional co-activator regulating gene expression during early development and hematopoiesis.^13, 14^ The KMT2A protein contains multiple conserved functional domains,^15^ and the SET domain is responsible for its histone H3 lysine 4 (H3K4) methyltransferase activity that mediates chromatin modifications associated with epigenetic transcriptional activation.^16, 17^ KMT2A is processed by taspase 1 into two fragments, MLL-C and MLL-N. These fragments re-associate and further assemble into different multiprotein complexes that regulate the transcription of specific target genes.^18, 19, 20^ It has been shown that aberrant chromosomal rearrangements of KMT2A generated the MLL-AF9 fusion protein that initiated murine acute myeloid leukemia.^21^ Other reports have shown that MLL fusion oncoprotein drive the expression of homeobox genes such as HOXA cluster genes and myeloid ecotropic viral integration site 1, which are known to induce leukemic transformation of hematopoietic progenitors and predict poor diagnosis for the disease.^22^ Furthermore, the expression of KMT2A is usually essential for the senescence-associated secretory phenotype,^23^ and KMT2A has been found to interact with the NF-*κ*B pathway to regulate brain cancer growth.^24^ In addition, new findings have demonstrated that KMT2A negatively regulates cell migration and invasion in cervical cancer.^25, 26^ However, the role of KMT2A in cancers other than MLL remains largely unknown.

Telomeres are repetitive (TTAGGG)_n_ DNA elements at the ends of chromosomes and shorten with each cell division, eventually leading to senescence (aging).^27, 28^ Telomerase adds the six-nucleotide repeating sequence onto the ends of chromosomes, providing a telomere maintenance mechanism for over 80% of human cancers. Human telomerase reverse transcriptase (hTERT) is the catalytic protein component of telomerase^29^ and expressed only in specific germ line cells, proliferative stem cells of renewal tissues, and cancer cells.^30, 31^ Overexpression of hTERT is a common feature of most human cancers believed to support cell immortalization.^32, 33, 34^ So far, it is known that hTERT is modulated at genetic, mRNA, protein and subcellular localization levels,^31^ and transcription factors including c-Myc, NF-*κ*B, STAT proteins and estrogen receptors^35^ have been reported to bind to the hTERT promoter to modulate its expression. The highly recurrent mutations in the promoter of TERT are found in over 50 cancer types, including papillary thyroid carcinoma,^36, 37^ hepatocellular carcinoma,^38^ epithelioid glioblastoma,^39^ bladder cancer,^40^ malignant pleural mesothelioma,^41^ melanoma,^42, 43, 44, 45^ are the most common mutation in many cancers. The newly described gremlin and recurrent somatic mutations in melanoma and other cancers in the TERT promoter, which create *de novo* E-twenty six/ternary complex factors (Ets/TCF) binding sites,^44, 45^ provide an insight into the possible cause of tumor-specific increased TERT expression. However, the precise mechanism behind the TERT activation in cancers remained unknown.

In our siRNA library screening, we identified a series of new proteins implicated in melanoma growth and progression. Among them, we chose KMT2A to evaluate its function in melanoma cell growth and apoptosis. Moreover, we explored the potential molecular mechanisms by which KMT2A regulated cell growth and its clinical significance. Our results showed that knockdown of KMT2A inhibited cell proliferation and induced apoptosis by activating the caspase-dependent signaling pathway, KMT2A promoted cell growth via hTERT signaling, and high expression of KMT2A was associated with poor prognosis in melanoma patients. Our study has not only revealed the role of KMT2A in melanoma progression for the first time, but also identified a potential therapeutic target for melanoma treatment.

## Results

### KMT2A knockdown inhibited cell proliferation in melanoma cells

**Figure 1:**
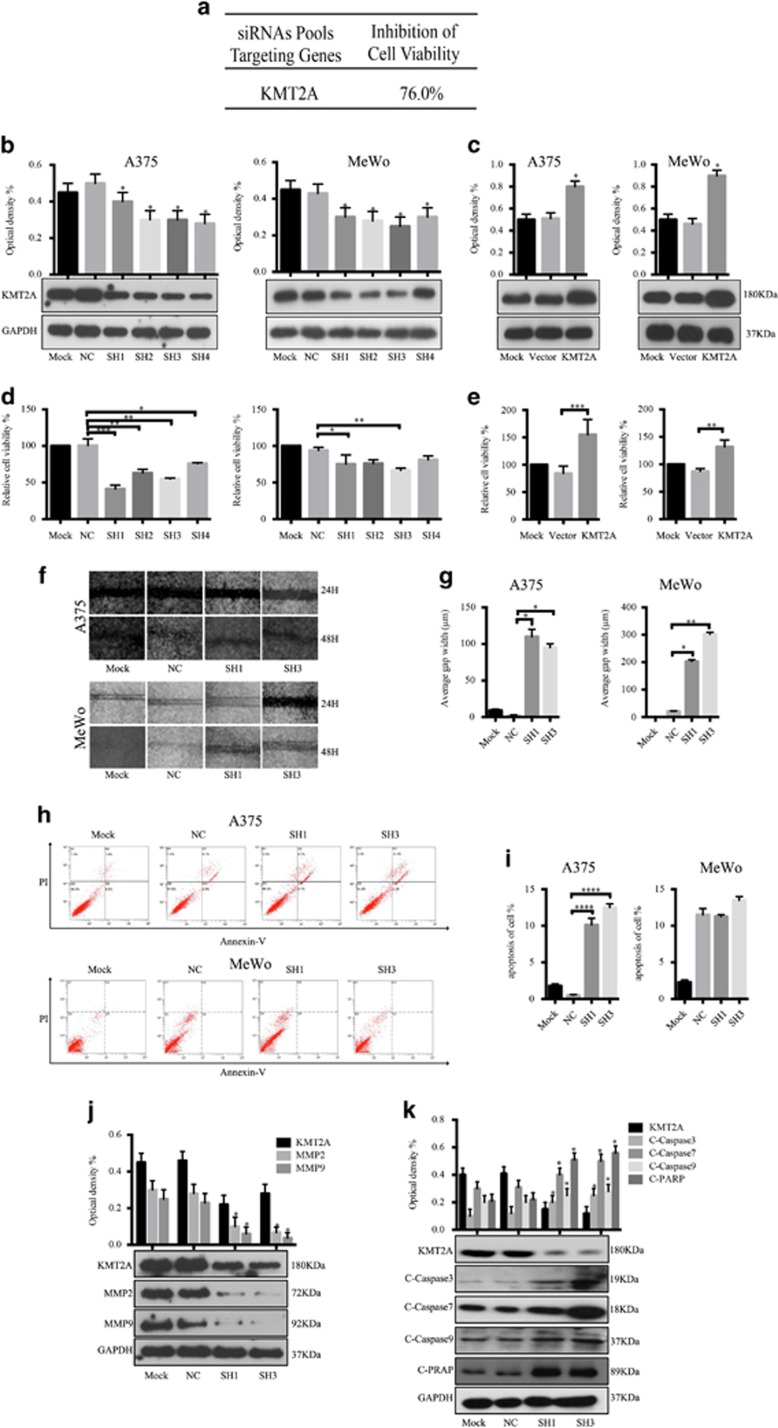
KMT2A was identified as a potential melanoma target. (**a**) A brief summary of the siRNA library screening results. (**b** and **c**) The KMT2A expression level was detected by western blot and the relative optical density % were analyzed in melanoma A375 and MeWo cells with KMT2A knockdown (**b**) or overexpression (**c**). (**d** and **e**) Viability of A375 and MeWo cells with KMT2A knockdown (**d**) or overexpression (**e**) was measured by MTS assay. (**f**) Migration ability of A375 and MeWo cells with KMT2A knockdown was measured by wound-healing assay and the average gap width (*μ*m) (**g**). (**e**) Apoptosis of A375 and MeWo cells with KMT2A knockdown was detected by FACS analysis and the relative apoptosis of cell % (**i**). (**j** and **k**) The expression of KMT2A, MMP2, MMP9 and the cleaved caspase3, caspase7, caspase9 and PARP proteins were detected by western blot in A375 cells with KMT2A knockdown

To discover and identify potential molecules and signaling pathways involved in the growth of melanoma, we screened a siRNA library targeting >6000 human genes in A375 melanoma cells and found that KMT2A knockdown by siRNA significantly suppressed the cell viability by 76.0% (Figure 1a), indicating that KMT2A, a transcriptional co-activator in cancer,^15, 46, 47^ could be a melanoma target.

To assess the role of KMT2A in melanoma cells, we first determined the expression levels of KMT2A in a panel of human melanoma cell lines (A375, MeWo, A431, WM35). The expression of KMT2A protein was detected by western blot, and the relative density were also analyzed (Figure 1b and c and Supplementary Fig. 1A, 1B). The results showed that knockdown of KMT2A by its specific shRNAs inhibited the expression of KMT2A (Figure 1b and Supplementary Fig. 1A), whereas overexpression of KMT2A markedly increased the expression of KMT2A (Figure 1c and Supplementary Fig. 1B).

To validate that KMT2A could promote melanoma cell proliferation; we measured its effect on the viability of human melanoma cell lines (A375, MeWo, A431, WM35) by MTS assay. Knockdown of KMT2A by its specific shRNAs significantly inhibited the viability of all the tested cell lines (Figure 1d and Supplementary Fig. 1C). In contrast, overexpression of KMT2A markedly increased the viability of the cells (Figure 1e and Supplementary Fig. 1D). Collectively, these results confirmed that KMT2A promoted melanoma cell growth.

### KMT2A Knockdown suppressed migration and induced apoptosis in melanoma cells

Next, we evaluated the effect of KMT2A knockdown on cell migration in A375 and MeWo cells by wound-healing assay. After 48 h, a scratch was made, the width of the gap or wounding space between cell layers remained distinct in cells treated with KMT2A-specific shRNAs, but the gap was almost fully occupied by the migrating cells in the control group (Figure 1f and g).

We further determined whether the inhibition of cell migration caused by KMT2A knockdown was associated with apoptosis. A375 and MeWo cells transfected with KMT2A-specific or scrabled control shRNAs for 48 h were harvested and subjected to apoptosis analysis by FACS. We found that a higher percentage of cells with KMT2A knockdown were apoptotic compared with the control (Figure 1h and i).

To illuminate the underlying molecular mechanism by which KMT2A knockdown inhibited cell migration and promoted apoptosis in melanoma, we analyzed the expression of a series of migration-related proteins and apoptosis-related proteins possibly affected by KMT2A. As shown in Figure 1j and k, KMT2A knockdown in A375 cells markedly decreased the expression of the migration markers MMP2 and MMP9, but increased the expression of cleaved-caspase3, cleaved-caspase7, cleaved-caspase9 and cleaved-PARP proteins. These results together indicated that knockdown of KMT2A in melanoma suppressed cell migration and induced apoptosis, which was at least partially mediated via the activation of the caspase-dependent signaling pathway.

### KMT2A bound to the promoter of hTERT to regulate its expression

Considering that KMT2A has been reported to regulate the expression of its target genes at their promoter regions, we examined whether it directly modulated the expression of genes involved in melanoma growth. hTERT opposes cellular senescence and is highly expressed in >90% of human cancers^48^ with a key role in melanoma progression.^49^ Therefore, we hypothesized that KMT2A promoted melanoma growth through regulating hTERT expression.

**Figure 2:**
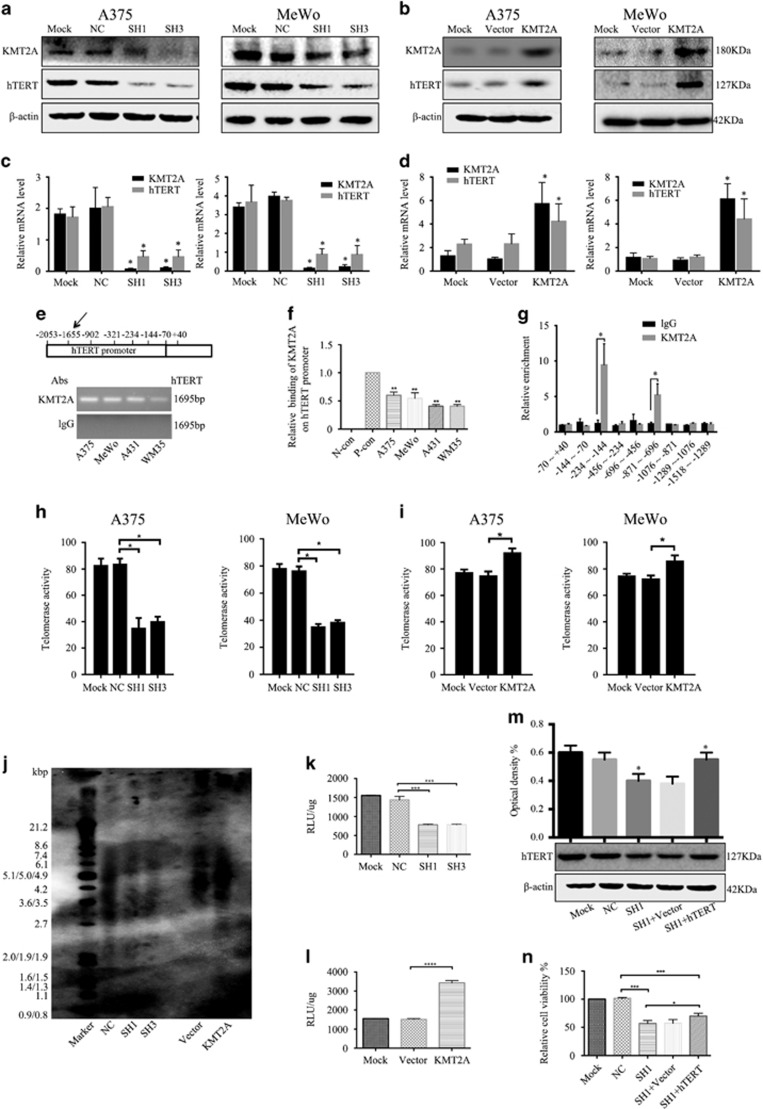
KMT2A bound to the promoter of hTERT to regulate its expression. (**a** and **b**) The protein levels of KMT2A and hTERT in A375 and MeWo cells with KMT2A knockdown (**a**) or overexpression (**b**) were analyzed by western blot. *β*-actin served as the loading control. (**c** and **d**) The mRNA expression of KMT2A and hTERT in A375 and MeWo cells with KMT2A knockdown (**c**) or overexpression (**d**) were detected by qRT-PCR. (**e**) ChIP was performed to detect the binding of KMT2A at the hTERT promoter. (**f**) Quantification of (**e**). P-con means the GAPDH primers, a positive control to demonstrate the efficacy of the EpiQuikTM Chromatin Immunoprecipitation Kit reagents and protocol. (**g**) ChIP-qPCR was performed to detect the binding of KMT2A at smaller fragments of hTERT promoter. (**h** and **i**) Telomerase activity in A375 and MeWo cells with KMT2A knockdown (**h**) or overexpression (**i**) was analyzed by Telo TAGGG Telomerase PCR ELISA assay kit. (**j**) The telomerase length in A375 cells with KMT2A knockdown and overexpression was analyzed by Telo TAGGG Telomerase length assay kit. (**k** and **l**) The activity of the hTERT promoter in A375 cells with KMT2A knockdown (K) or overexpression (**l**) was measured by dual-luciferase assay. (**m**) hTERT expression was detected by western blot in A375 cells with KMT2A knockdown alone or together with hTERT overexpression. (**n**) MTS assay was performed to measure the viability of the cells in (**m**)

We first assessed the regulation of hTERT protein and mRNA expression by KMT2A in melanoma cells by western blot and qRT-PCR analysis. As shown in Figure 2a and c, the protein and mRNA levels of hTERT were reduced when KMT2A was silenced, but were significantly increased when KMT2A was overexpressed (Figure 2b and d), supporting that KMT2A could regulate melanoma growth via hTERT signaling.

The regulation of hTERT expression occurs through multiple avenues including CpG promoter methylation,^50^ alternative splicing^51^ and so on, and transcriptional regulation is the limiting step.^52^ A number of factors have been identified to regulate the activity of the hTERT promoter directly or indirectly.^53, 54, 55^ To test if KMT2A anchored at the hTERT promoter to regulate its expression, ChIP assay was performed in melanoma cells. The results showed that the hTERT promoter region was amplified by PCR from the complexes immunoprecipitated by the antibody against KMT2A, but not from the complexes precipitated by the IgG negative control (Figure 2e and f).

Mutations in the TERT promoter have been found in melanoma^44, 45^ and are known to regulate TERT expression. To determine whether KMT2A bind to the segment of hTERT promoter associated with mutations, ChIP-qPCR assay was performed. The smaller hTERT promoter fragments were amplified by PCR. As shown in Figure 2g, the fragments −234~−144 and −871~−696 were more enrich than the other fragments, especially the −871~−696 fragment, indicating that these regions maybe the regions that KMT2A bound on the promoter of hTERT.

Next, we investigated the effect of KMT2A on the hTERT promoter activity. The luciferase reporter assay showed that knockdown of KMT2A significantly decreased the hTERT promoter activity in A375 cells compared with the non-silencing shRNA control (Figure 2k), whereas overexpression of KMT2A markedly enhanced the hTERT promoter activity (Figure 2l). Taken together, these data indicated that KMT2A promoted hTERT expression at the transcriptional level through binding to its promoter in melanoma cells.

We also investigated the effect of KMT2A on telomerase activity and telomerase length. The ELISA assay showed that knockdown of KMT2A significantly decreased the telomerase activity in A375 and MeWo cells (Figure 2h), whereas overexpression of KMT2A markedly enhanced the telomerase activity (Figure 2i). The Telo TAGGG Telomere Length Assay showed that knockdown of KMT2A significantly decreased the telomerase length in A375 cells compared with the non-silencing shRNA control (Figure 2j), whereas overexpression of KMT2A markedly enhanced the telomerase length (Figure 2j). These data indicated that KMT2A regulated telomerase activity and telomerase length through binding to hTERT promoter in melanoma cells.

In addition, to determine whether knockdown of KMT2A inhibited melanoma cell growth through regulating hTERT expression, MTS assay was performed to explore if overexpression of hTERT could offset the inhibitory effect caused by KMT2A knockdown. As shown in Figure 2m and n, cell viability was inhibited when KMT2A was silenced, but this inhibition was partly rescued by hTERT overexpression in A375 cells, confirming that KMT2A controlled cell growth through hTERT.

### KMT2A regulated cancer stem cell marker expression and tumorsphere formation through hTERT signaling

**Figure 3:**
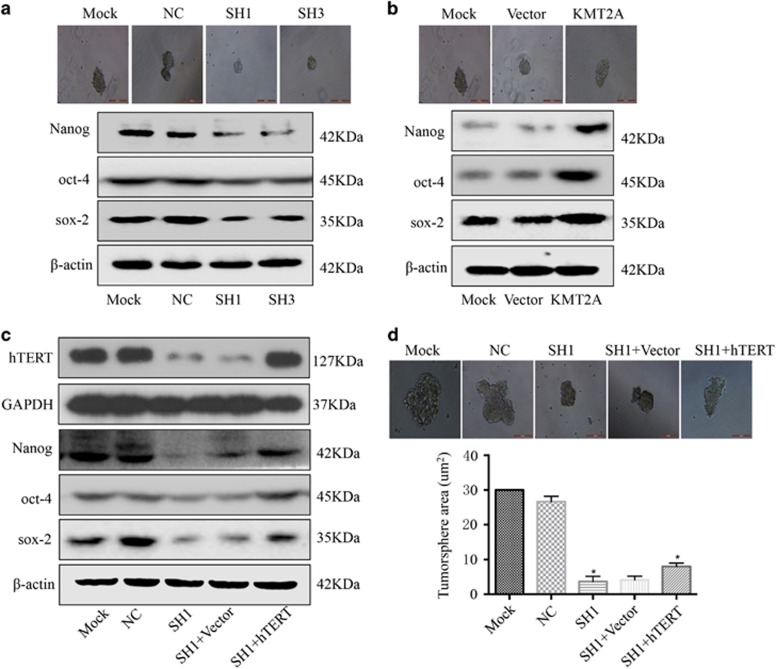
KMT2A regulated cancer stem cell marker expression and tumorsphere formation through hTERT signaling. (**a** and **b**) Human melanoma A375 cells were transfected with KMT2A shRNAs or KMT2A overexpression plasmid. After 48 h of transfection, the levels of Nanog, oct-4 and sox-2 were analyzed by western blot. *β*-actin served as the loading control. Also, the tumorsphere formation ability was tested, and representative images were displayed. (**c**) hTERT, Nanog, oct-4 and sox-2 expression was detected by western blot in A375 cells with KMT2A knockdown alone or with KMT2A knockdown and hTERT overexpression. (**d**) The tumorsphere formation ability in the cells in (**c**) was examined. Cells with representative morphology were shown. For quantification, more than 100 cells were inspected per experiment

Accumulating evidence suggests that hTERT is associated with the characteristics of caner stem cells.^56, 57, 58^ Given that hTERT had a role in tumorsphere formation, we examined if KMT2A could promote the stemness of melanoma cells through the hTERT signaling pathway. The tumorsphere formation assay in A375 cells showed that knockdown of KMT2A markedly decreased the expression of the stem cell markers Nanog, oct-4 and sox-2, and significantly repressed the tumorsphere formation ability of cells (Figure 3a). In contrast, overexpression of KMT2A increased the expression of Nanog, oct-4 and sox-2, and enhanced the tumorsphere formation ability of cells (Figure 3b). Moreover, overexpression of hTERT partially reversed the tumorsphere formation inhibition caused by KMT2A knockdown (Figure 3c and d), suggesting that KMT2A regulated the expression of cancer stem cell markers and tumorsphere formation in melanoma via the hTERT signaling pathway.

### KMT2A knockdown inhibited melanoma progression in a mouse xenograft model

**Figure 4:**
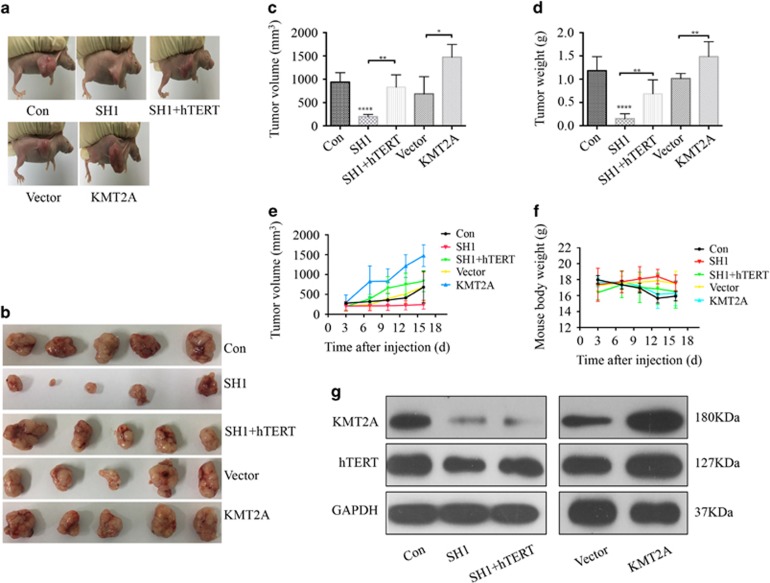
KMT2A knockdown inhibited melanoma progression in a mouse xenograft model. Control shRNA (Con), KMT2A-shRNA (SH1), KMT2A-shRNA+hTERT overexpression (SH1+hTERT), empty vector (Vector) and KMT2A overexpression plasmid (KMT2A) were intratumorally injected into mice. (**a**) Representative photographs of the tumor bearing mice. (**b**) Morphology of tumor xenograft from each mouse. (**c**) Tumor volume of each mouse at the time of sacrifice. (**d**) Tumor weight of each mouse at the end of the experiment. (**e**) Tumor volume of each mouse was measured and recorded every three days through the course of the experiment. (**f**) Body weight of each mouse was monitored. (**g**) The expression of KMT2A and hTERT in tumor xenografts were tested by western blot

The oncogenic role of KMT2A in melanoma was further examined in a mouse xenograft model. A375 cells (5 × 10^6^ in 100 *μ*l PBS) were injected subcutaneously into the left flank of female athymic nude mice aged 3–4 weeks. When the formed tumor reached 100 mm^3^, the animals were randomly divided into five groups (five per group) and, respectively, intratumorally injected with the control shRNA, KMT2A shRNA, KMT2A shRNA+hTERT overexpression, vector and KMT2A overexpression once every three days for six times. After administration for 18 days, both the tumor volumes (Figure 4c and e) and tumor weight (Figure 4d and f) were measured. As shown in Figure 4a and e, knockdown of KMT2A markedly suppressed melanoma tumor growth in size, volume and weight, whereas overexpression of KMT2A promoted tumor growth. Furthermore, hTERT overexpression remarkably reversed the growth inhibition caused by KMT2A knockdown. In addition, all these treatments did not significantly affect the body weight of the mice (Figure 4f), and no other signs of acute or delayed toxicity were observed in the mice during treatment.

Furthermore, the results from western blot analysis of tissue lysates of the xenograft tumors demonstrated that co-treatment with KMT2A-shRNA1 and hTERT plasmid in the mice enhanced the expression of hTERT, which was suppressed when treatment with KMT2A-shRNA1 (Figure 4g). The HE staining also showed that the tumor cells in the nonspecifically shRNA-treated control mice were irregular and had abundant cytoplasm, and large and deformity nuclei (data were not shown). Moreover, the expression of KMT2A and hTERT was determined by immunohistochemical (IHC) staining. Consistent with the *in vitro* results (Figure 2a and g), knockdown of KMT2A significantly decreased the levels of KMT2A and hTERT. All these results from the xenograft model indicated that KMT2A knockdown indeed had anti-cancer effects in melanoma, which were at least partially achieved by activating the hTERT signal pathway.

### Expression of KMT2A and hTERT was positively correlated in melanoma patient samples

**Figure 5:**
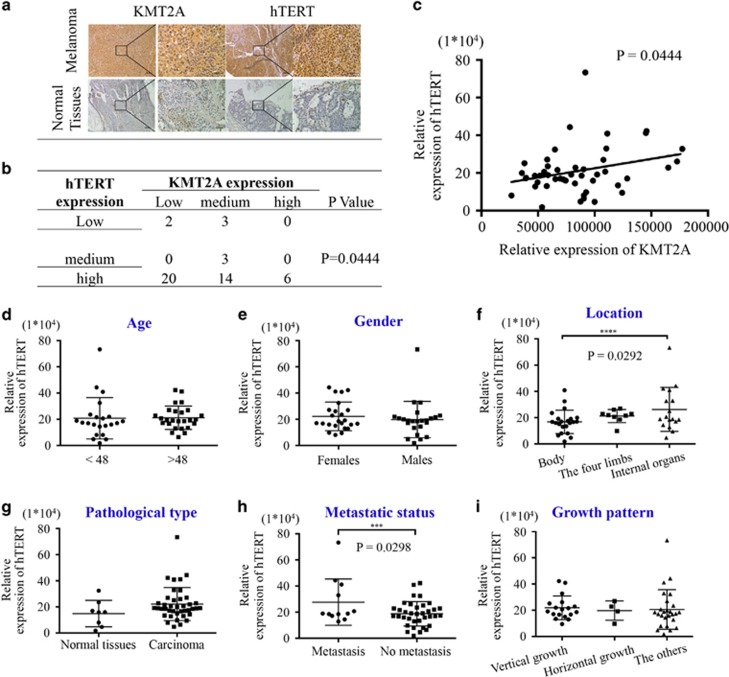
KMT2A expression was positively correlated with hTERT expression in melanoma patient tissues. (**a**) Representative images of the immunohistochemical staining of KMT2A and hTERT in human normal and melanoma tissues. 200 × and 400 × magnification. (**b** and **c**) The correlation between the expression of KMT2A and hTERT in human melanoma tissues from 48 patients. (**d**–**i**) Correlation analyses of hTERT protein expression in relation to different clinicopathologic variables in melanoma patient tissues

To uncover the clinical significance of KMT2A and further confirm its relevance with hTERT, we detected their expression in melanoma tissue samples. The level of KMT2A expression was categorized into three classes (high, moderate and low), and the representative staining images of KMT2A and hTERT in melanoma and normal tissues were shown in Figure 5a. Among the 48 samples tested (Supplementary Table 1), 6 (12.5%) had high KMT2A expression and 22 (45.8%) had low KMT2A expression, whereas 40 (83.3%) had high hTERT expression and 5 (10.4%) had low hTERT expression (Figure 5b). Correlation analysis showed that the expression of KMT2A and hTERT were positively correlated in human melanoma (*P*=0.0444) (Figure 5c). Also, the relationships between hTERT expression and different clinicopathologic variables were analyzed (Figure 5d and i), and the expression of hTERT was significantly correlated with the location (*P*=0.0292) (Figure 5f) and the metastatic status of the melanoma tissues (*P*=0.0298) (Figure 5h).

### High expression of KMT2A predicted poor prognosis in melanoma patients

**Figure 6:**
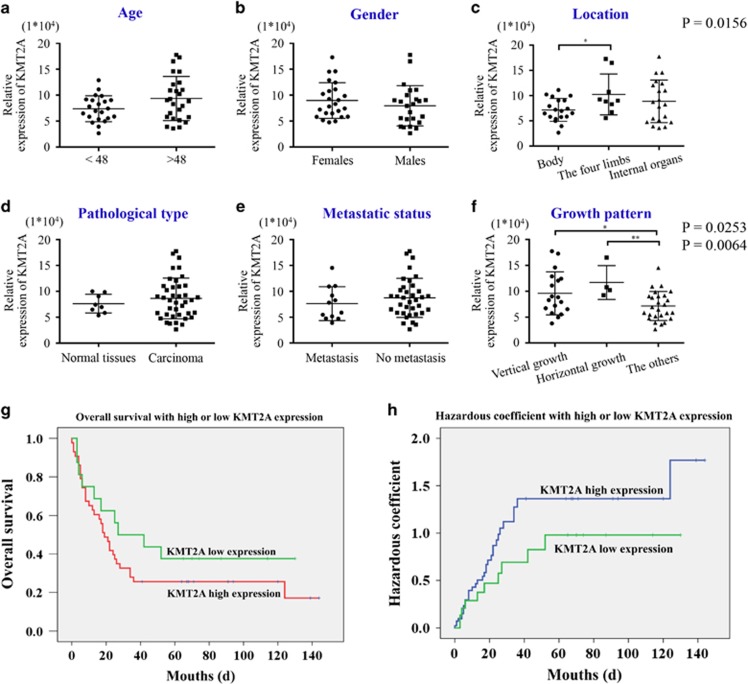
High expression of KMT2A predicted poor prognosis in melanoma patients. (**a**–**f**) Correlation analyses of KMT2A protein expression in relation to different clinicopathologic variables in 48 melanoma tissue samples. (**g**) Kaplan–Meier analysis showed high overall survival of melanoma patients with low KMT2A expression. (**h**) Hazard analysis showed high KMT2A expression predicted low overall survival of melanoma patients

Analysis of the relationships between KMT2A expression and different clinicopathologic variables (Figure 6a and f) showed that the expression of KMT2A was significantly correlated with the location (*P*=0.0156) (Figure 6c) and the growth pattern of melanoma (*P*=0.0253 and 0.0064) (Figure 6f). We further investigated the prognostic role of KMT2A in melanoma patients. The OS analysis indicated that the patients with KMT2A low expression had a significantly higher survival rate compared with those with KMT2A high expression (Figure 6g), which was supported by the hazardous coefficient analysis (Figure 6h). Collectively, our clinical results validated the protumorigenic function of KMT2A in melanoma, and suggested that it was mediated through the hTERT signaling pathway.

## Discussion

In the past 20 year, much progress has been made in understanding the structure and biological functions of the KMT2A protein.^15, 59^ However, very few studies have explored the functions of KMT2A in malignant melanoma. In this study, we have demonstrated the functional significance of KMT2A in melanoma progression *in vitro* and *in vivo*. KMT2A was identified as a candidate target from a siRNA library screening, and we found that KMT2A knockdown inhibited cell proliferation, promoted apoptosis and suppressed the growth of melanoma xenograft. Our further analysis showed that KMT2A knockdown suppressed the expression of MMP2, MMP9 and promoted the cleavage of Caspase3/Caspase7/Caspase9 and PARP in melanoma cells, and repressed the expression of hTERT and the Nanog/oct-4/sox-2 stem cell markers. Importantly, we uncovered that KMT2A bound at the promoter of hTERT to regulate its expression (Supplementary Fig. 2). Overexpression of hTERT partially reversed the inhibitory effects caused by KMT2A knockdown on melanoma growth and tumorsphere formation, suggesting that KMT2A promoted melanoma growth in part via the hTERT pathway. In addition, our clinical data indicated that KMT2A and hTERT were positively correlated and KMT2A high expression predicted poor prognosis in melanoma patients. To the best of our knowledge, it is the first study to document the role and molecular mechanism of KMT2A in melanoma carcinogenesis and development.

hTERT encodes a rate-limiting catalytic subunit of telomerase that maintains genomic integrity.^60^ In our study, ChIP assay showed that KMT2A bound to the promoter of hTERT (−1655~+40) in melanoma cell lines. Recurrent and mutually exclusive C>T or CC>TT transition mutations were identified in the promoter region of the reverse transcriptase catalytic subunit of the telomerase gene (TERT) in melanoma, suggesting that they enhanced the expression of TERT gene and played important roles in the melanoma pathogenesis.^42^ We also determined whether KMT2A bind to the segment of hTERT promoter associated with mutations. The results from ChIP-qPCR showed that the fragments −234~−144 and –871~−696 were more enrichment than the other fragments, especially the −871~−696 fragment. These results were not the same as the previous studies. The most recurrent melanoma nucleotide substitution has been shown to include BRAF (chr7, 140,453,136A>T V600E), NRAS (chr1, 115, 256, 529T>C Q61R) and TERT core promoter mutations (C228T and C250T),^44^ but the precise mechanism behind the TERT activation in cancers remains largely unknown and need to be studied in the future.

Telomerase has a pivotal role in cancer by maintaining the ends of chromosomes and the potential for unlimited proliferation in cells.^48^ As the catalytic subunit of telomerase, hTERT determines the activity of telomerase.^61^ Multiple transcription factor binding sites exist in the promoter region of hTERT, and its expression is tightly controlled by transcriptional activactors^62, 63^ and tumor suppressors.^35^ Besides, methylation of the histones at the hTERT promoter region recruits histone acetyl transferase and promotes hTERT transcription.^64^ Nonetheless, it remains unclear how hTERT is regulated in melanoma.

KMT2A is a transcriptional co-activator with H3K4 methyltransferase activity.^16^ Its genetic rearrangements often result in ontogenetic fusion proteins causing acute leukemia in pediatric and adult patients.^65, 66, 67^ Here, we discovered that KMT2A regulated the expression of hTERT by binding to its promoter. Further investigation is needed to determine whether KMT2A activates hTERT by methylating the histones at its promoter, and whether the activation of hTERT is caused by a novel mechanism other than the chromosomal translocations of KMT2A.

In summary, our study has revealed a potential oncogenic role of KMT2A in melanoma. Our results demonstrated that KMT2A bound at the promoter of hTERT to activate its expression, and thus promoted melanoma growth through the hTERT pathway in cell lines and a xenograft mouse model. Furthermore, high expression of KMT2A was related to poor prognosis in melanoma patients, suggesting that KMT2A could be a potential biomarker for the diagnosis and a therapeutic target for the treatment of melanoma in the future.

## Materials and methods

### Cell lines and cell culture

Human melanoma cell lines A375, MeWo, A431 and WM35 were from ATCC, cultured in DMEM with 10% FBS, 100 *μ*g/ml penicillin and 100 *μ*g/ml streptomycin, and maintained in standard culture condition.

### Reagents and antibodies

GAPDH and *β*-actin antibodies were from Proteintech (Rosemont, IL, USA). KMT2A, MMP2, MMP9, cleaved-caspase3, cleaved-caspase7, cleaved-caspase9, cleaved-PARP, Nanog, oct-4 and sox-2 antibodies were from Cell Signaling Technology (Beverly, MA, USA). hTERT antibodies were from Santa Cruz Biotechnology (Santa Cruz, CA, USA) and Novus NB110-89471.

### Plasmid vectors

The plasmid pMSCV-FlagMLL-pl-ENL (5613) (KMT2A) and its corresponding vector were gifts from Robert Slany (Addgene plasmid # 20873). Recombinant plasmid pGL3-hTERT-438 expressing luciferase driven by an hTERT promoter (−1655 to +40) was constructed in our laboratory.

### shRNA design

The shRNAs (see Supplementary Table 2 for sequences) were purchased from Shanghai GenePharma Company (Shanghai, China).

### Cell viability assay

Cell viability was determined using the MTS assay (Roche Diagnosis, Indianapolis, IN, USA).

### Wound-healing assay

Wound-healing assay was used to detect cell migration ability and performed as described in ref. 68.

### Apoptosis assay

Apoptosis assay was performed as described in ref. 69.

### Nuclear extraction

Nuclear extraction was done as described in ref. 70.

### Chromatin immunoprecipitation (ChIP) assay

ChIP experiment was performed using EpiQuikTM Chromatin Immunoprecipitation Kit (Bsae Catalog # P-2002) according to the manufacturer’s instructions, and the primers for PCR amplification of the hTERT promoter are in Supplementary Table 2.

### Telomerase activity assay

Telomerase activity was analyzed by Telo TAGGG Telomerase PCR enzyme-linked immunosorbent assay kit (Roche, REF 11854666910) according to the manufacturer’s instructions.

### Telomerase length assay

Telomere length was measured by a highly sensitive nonradioactive chemiluminescence assay utilizing southern blot analysis of terminal restriction fragments obtained by digestion of genomic DNA with frequently cutting restriction enzymes (*Hin*fI/*Rsa*I) with a Telo TAGGG telomere length assay kit (Roche, REF 12209136001) according to the manufacturer’s instructions. Telomeric smears were revealed by exposure on X-ray film.

### Promoter reporters and dual-luciferase assay

The core promoter region of hTERT (−1655 to +40) was inserted between the *Sac*I and *Hin*dІІІ sites of the firefly luciferase vector pGL4.10 (Promega, Madison, WI, USA), and Renilla luciferase reporter vector pRL-TK was used as a control. Dual-luciferase assay was performed as described in ref. 68 and the luciferase activity was measured using the Dual-Luciferase Reporter Assay System (Promega).

### Western blot

Western blot was performed as described in ref. 68.

### Real time PCR (qPCR)

SYBR Green PCR master mix (Toyobo Life Science, Shanghai, China) was used for qPCR, followed by detection with a Bio-Rad CFX96 and analyzed with the Bio-Rad Manager software (Bio-Rad, Hercules, CA, USA). The PCR primers were synthesized by TaKaRa and were purchased from GeneCopoeia (KMT2A Hs-QRP-33581, TERT Hs-QRP-22639, GAPDH Hs-QRP-20169) (see Supplementary Table 2 for sequences).

### Animal experiment and tissue processing

All animal procedures were performed in accordance with the Guide for the Care and Use of Laboratory Animals (NIH publications Nos. 80-23, revised 1996) and the Institutional Ethical Guidelines for Animal Experiments developed by Sun Yat-sen University. A375 cells (5 × 10^6^ in 100 *μ*l PBS) were injected subcutaneously into the left flank of female athymic nude mice aged 3–4 weeks. When the formed tumor reached 100 mm^3^, the animals were randomly divided into five groups (five per group) and, respectively, intratumorally injected with control shRNA, KMT2A shRNA, KMT2A shRNA+hTERT overexpression, vector and KMT2A overexpression once every three days for six times. The tumor size was measured using a Vernier caliper and the tumor volume was calculated as *V*=(length × width × height)/2. The experiment was terminated 22 days after tumor cell inoculation. The mice were then killed and the tumors were excised and weighed.

Tumor tissues from the above treated animals were processed as described in ref. 68.

### Melanoma tissue microarray and IHC assay

Tissue microarray for KMT2A and hTERT expression was purchased from Fanpu Biotech, Inc. (Guilin, China), with 48 melanoma tissues from patients without anti-cancer treatments. Clinicopathologic information was documented for all cases. Tissue microarray for KMT2A alone was purchased from Novus Biologicals (Littleton, CO, USA), with 59 melanoma tissues from patients without anti-cancer treatments. OS was documented for all cases. IHC staining for the microarray slides was performed as described in ref. 68.

### Statistical analysis

Each experiment was done three times and the results were presented as the mean±s.d. GraphPad Prism was used for statistical analysis. Student’s *t*-test was used and *P<0.05, ***P<0.001, and ****P<0.0001 indicated significant difference.
