# Supplementary figures and images for: KMT2A promotes melanoma cell growth by targeting hTERT signaling pathway
Source: Cell Death Dis. 2017 Jul 20;8(7):e2940–. doi: 10.1038/cddis.2017.285 (PMC5550845; doi:10.1038/cddis.2017.285)

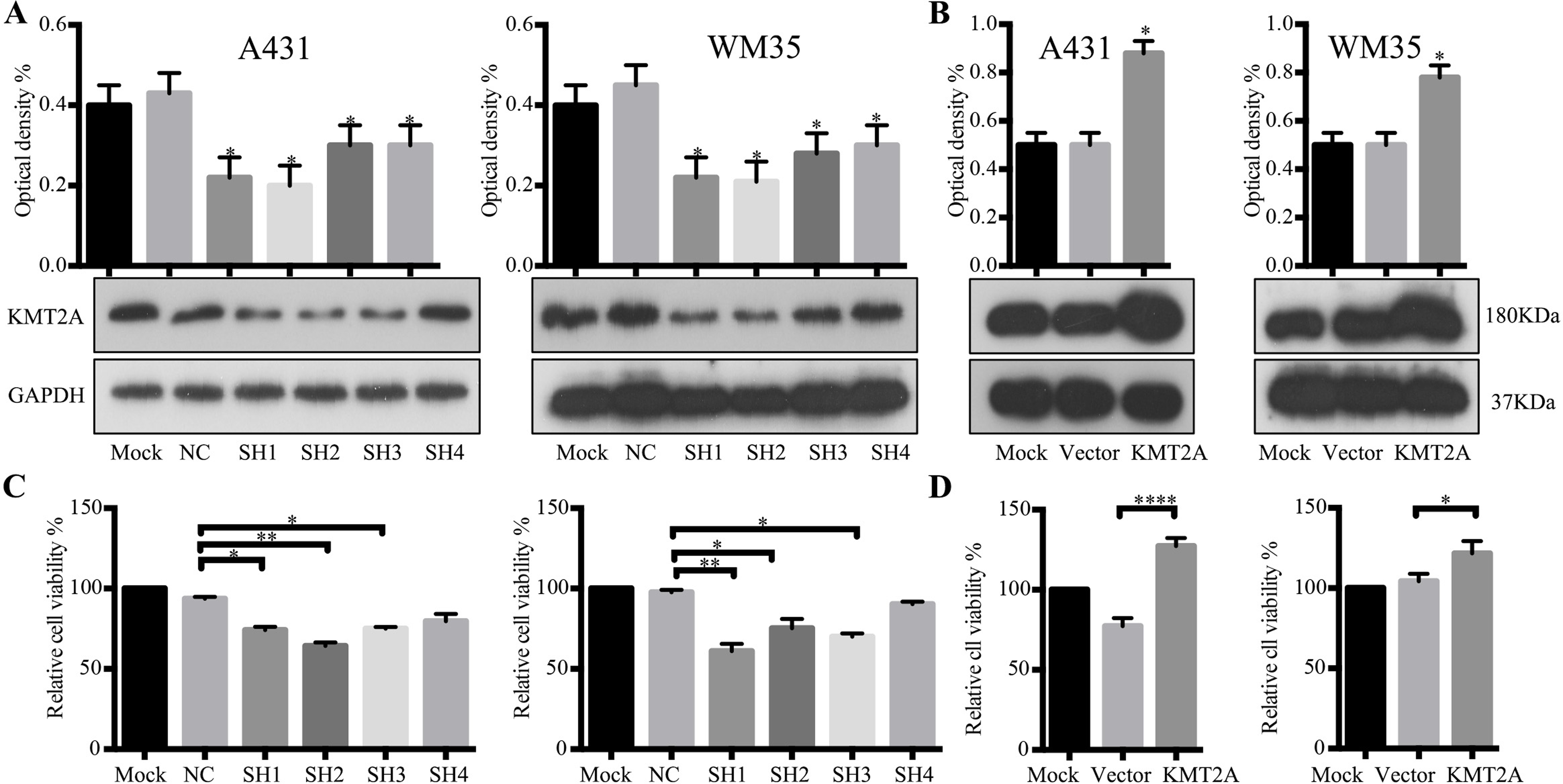

Supplement: Supplementary Figure 1 [file cddis2017285x1.tif]

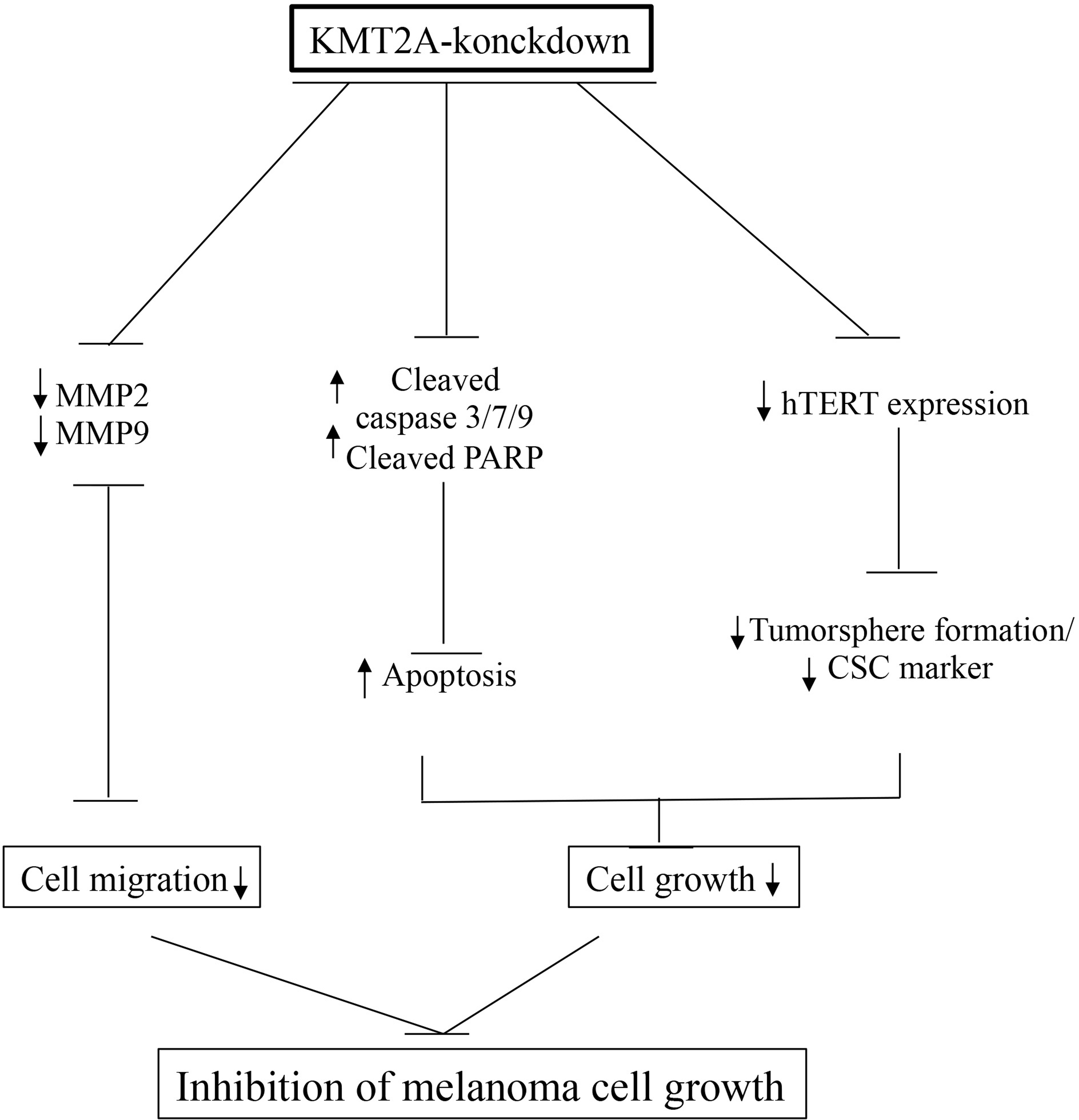

Supplement: Supplementary Figure 2 [file cddis2017285x2.tif]

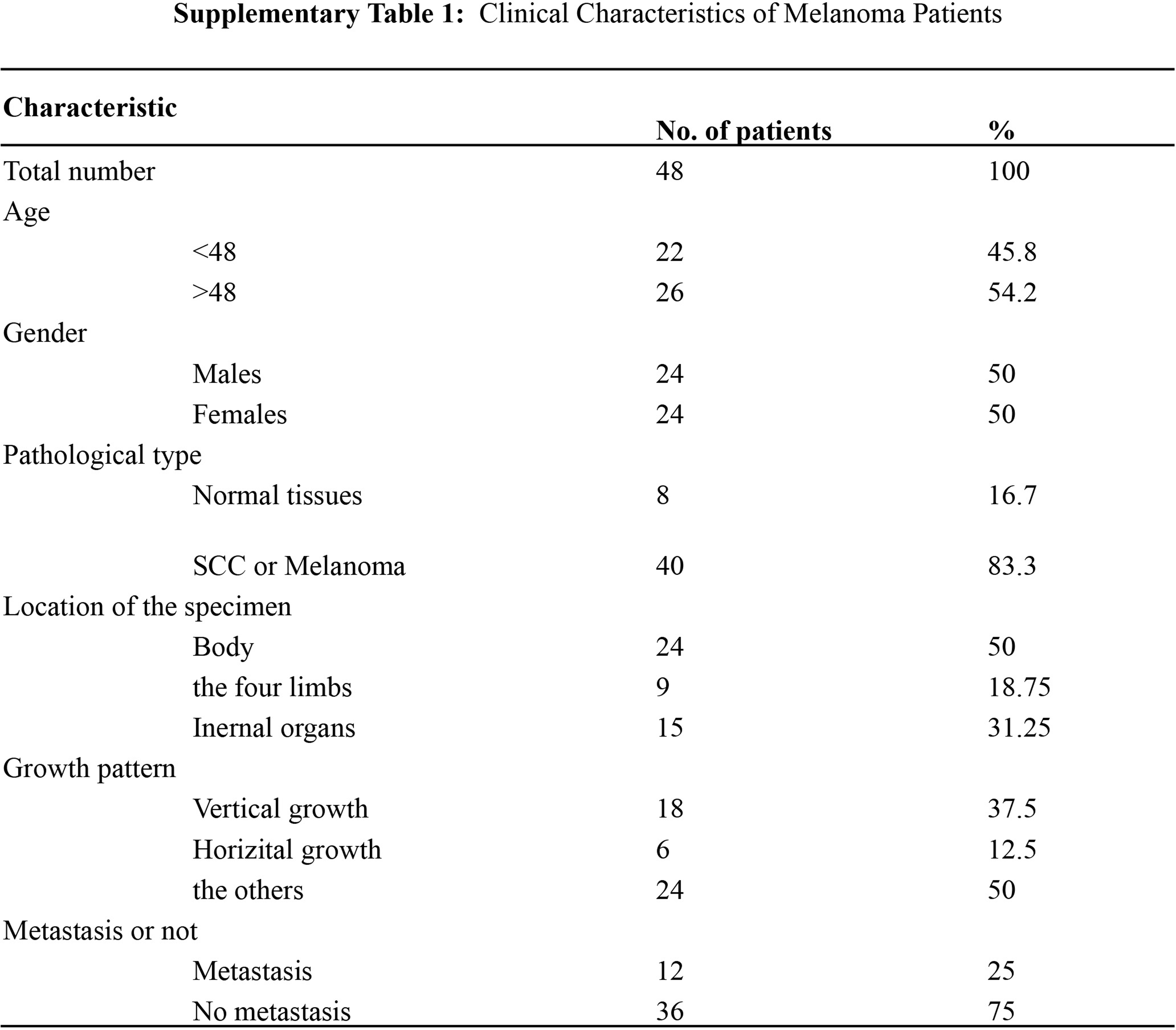

Supplement: Supplementary Table 1 [file cddis2017285x3.tif]
