# Supplementary material for: KMT2A promotes melanoma cell growth by targeting hTERT signaling pathway
Source: Cell Death Dis. 2017 Jul 20;8(7):e2940–. doi: 10.1038/cddis.2017.285 (PMC5550845; doi:10.1038/cddis.2017.285)
Supplement: Supplementary Table 2 [file cddis2017285x4.docx]

**Supplementary Table 2: shRNA and PCR Primer Sequences**

| Name | | Sequence |
| --- | --- | --- |
| KMT2A knockdown | KMT2A-shRNA1 forward | 5’-CCGGGCACTGTTAAACATTCCACTTCTCGAGAAGTGGAATGTTTAACAGTGCTTTTTG-3’ |
|  | KMT2A-shRNA1 reverse | 5’-AATTCAAAAAGCACTGTTAAACATTCCACTTCTCGAGAAGTGGAATGTTTAACAGTGC-3’ |
|  | KMT2A-shRNA2 forward | 5’-CCGGGCCTCCATCAACAGAAAGGATCTCGAGATCCTTTCTGTTGATGGAGGCTTTTTG-3’ |
|  | KMT2A-shRNA2 reverse | 5’-AATTCAAAAAGCCTCCATCAACAGAAAGGATCTCGAGATCCTTTCTGTTGATGGAGGC-3’ |
|  | KMT2A-shRNA3 forward | 5’-CCGGCCCATCCAGAACCAGAAGTATCTCGAGATACTTCTGGTTCTGGATGGGTTTTTG-3’ |
|  | KMT2A-shRNA3 reverse | 5’-AATTCAAAAACCCATCCAGAACCAGAAGTATCTCGAGATACTTCTGGTTCTGGATGGG-3’ |
|  | KMT2A-shRNA4 forward | 5’-CCGGCCGGTCAATAAGCAGGAGAATCTCG AGCTCGAGTTTTTG-3’ |
|  | KMT2A-shRNA4 reverse | 5’-AATTCAAAAACCGGTCAATAAGCAGGAGAATCTCGAGATTCTCCTGCTTATTGACCG-3’ |
| ChIP | hTERT promoter forward | 5’-TGGCCCCTCCCTCGGGTTAC-3’ |
|  | hTERT promoter reverse | 5’-TGAAGGGGCAGGACGGGTGC-3’ |
| qPCR | hTERT forward | 5’-TCACAGGCTTCCATTGACCAG-3’ |
|  | hTERT reverse | 5’-CCGAGGCTTTTCTACCAGA-3’ |
|  | GAPDH forward | 5’-AATCCCATCACCATCTTCC-3’ |
|  | GAPDH reverse | 5’-CATCACGCCACAGTTTCC-3’ |
| CHIP-qPCR | hTERT-1518-forward | 5’-GCATAATCTTCTGCTTCCAT-3’ |
|  | hTERT-1289-forward | 5’-TACAAGACGAGGCTAACCTCC-3’ |
|  | hTERT-1289-reverse | 5’-GCTCAAGTTTGGATCTAAGGG-3’ |
|  | hTERT-1076-forward | 5’-CCCTTAGATCCAAACTTGAGCAACC-3’ |
|  | hTERT-1076-reverse | 5’-AACATCTGGTCACATCCCGCCC-3’ |
|  | hTERT-871-forward | 5’-GGGCGGGATGTGACCAGATGTT-3’ |
|  | hTERT-871-reverse | 5’-CAGGTTCTCAGGCGGCGAG-3’ |
|  | hTERT-696-forward | 5’-CTCGCCGCCTGAGAACCTG-3’ |
|  | hTERT-696-reverse | 5’-CGGAGACCCAGGGCTGCC-3’ |
|  | hTERT-456-forward | 5’-GGCAGCCCTGGGTCTCCG-3’ |
|  | hTERT-456-reverse | 5’-CCTGGCCCCGACAGCGCAGC-3’ |
|  | hTERT-234-forward | 5’-GCTGCGCTGTCGGGGCCAGG-3’ |
|  | hTERT-234-reverse | 5’-GGCAGGACGGGTGCCCGGGT-3’ |
|  | hTERT-144-forward | 5’-ACCCGGGCACCCGTCCTGCC-3’ |
|  | hTERT-144-reverse | 5’-GCTGGGCCGGGGACCCGGGA-3’ |
|  | hTERT-70-forword | 5’-TCCCGGGTCCCCGGCCCAGC-3’ |
|  | hTERT-70-reverse | 5’-CCAGGGCTTCCCACGTGCGC-3’ |
|  | hTERT+40-reverse | 5’-CCCATCGGCCAGGGCTTCCCACG-3’ |
