# Supplementary material for: KMT2A promotes melanoma cell growth by targeting hTERT signaling pathway
Source: Cell Death Dis. 2017 Jul 20;8(7):e2940–. doi: 10.1038/cddis.2017.285 (PMC5550845; doi:10.1038/cddis.2017.285)
Supplement: Supplementary Figures and Tables Legends [file cddis2017285x5.docx]

**Supplementary Figures and Tables Legends**

**Supplementary Figure 1. The effect of KMT2A on cell viability.** (A and B) The KMT2A expression level was detected by Western blot and the relative optical density % was analyzed in melanoma A431 and WM35 cells with KMT2A knockdown (A) or overexpression (B). (C and D) Viability of A431and WM35 cells with KMT2A knockdown (C) or overexpression (D) was measured by MTS assay.

**Supplementary Figure 2. The mechanism of KMT2A knockdown inhibiting melanoma growth via hTERT in human.** The schematic diagram illustrates the molecular mechanism by which KMT2A regulates melanoma growth. Upward symbol (↑) indicated positive regulation. The downward arrow (↓) indicated negative regulation.

**Supplementary Table 1: The clinical characteristics of melanoma patients.** The clinical characteristics of the 48 patients were analyzed.

**Supplementary Table 2: shRNA and PCR Primer Sequences.** All the shRNAs and PCR primers used in this study are listed with their sequences.
